# IS*256*-Mediated Overexpression of the WalKR Two-Component System Regulon Contributes to Reduced Vancomycin Susceptibility in a *Staphylococcus aureus* Clinical Isolate

**DOI:** 10.3389/fmicb.2019.01882

**Published:** 2019-08-14

**Authors:** Makoto Kuroda, Tsuyoshi Sekizuka, Hidehito Matsui, Jun Ohsuga, Toshio Ohshima, Hideaki Hanaki

**Affiliations:** ^1^Pathogen Genomics Center, National Institute of Infectious Diseases, Shinjuku, Japan; ^2^Infection Control Research Center, Kitasato University, Minato-ku, Japan; ^3^Department of Clinical Laboratory, Tokai University Oiso Hospital, Kanagawa, Japan; ^4^Department of Medical Risk and Crisis Management, Chiba Institute of Science, Chiba, Japan

**Keywords:** glycopeptide, vancomycin intermediate resistance, genome-wide SNVs, IS*256*, *walKR*, lytic enzyme

## Abstract

Vancomycin (VAN)-intermediate-resistant *Staphylococcus aureu*s (VISA) is continually isolated globally, with a systematic review suggesting a prevalence of 2% in all blood culture samples. Most VISA strains exhibit common characteristics, such as a thickened cell wall, reduced autolysis, and attenuated virulence. Here, based on multi-omics approaches, we have characterized clinical VISA isolates obtained through prolonged antimicrobial treatment in a single patient. All VISA isolates were isogenic, based on multi-locus sequence typing (MLST) ST5, SCC*mec* type II (2A), and *spa* type t17639. Core-genome single nucleotide variations (SNVs) found among thirteen isolates during the patient’s hospitalization, indicated clonality, but not notable genetic features of the VISA phenotype. We determined the complete genome sequence of VAN-susceptible strain KG-03 (minimum inhibitory concentration [MIC] 0.5 μg/mL) and two VISA strains, KG-18 and KG-22 (MIC 8.0 and 4.0 μg/mL, respectively). Comparative genome analysis showed remarkable strain-specific IS*256* insertions. RNA-Seq transcriptome analysis revealed IS*256*-mediated overexpression of the *walKR* two-component system in VISA KG-18, possibly leading to modulation of cell wall integrity (*lytM* and *sceD*) and surface charge (*mprF* and *dltABCD*). In addition, secretome analysis indicated that cell wall-anchored proteins (Protein A, SasG, and SdrD) were significantly decreased. KG-18 and KG-22 exhibit thickened cell wall, and are relatively resistant to lysostaphin, which cleaves a staphylococcus-unique pentaglycine chain in the peptidoglycan. We conclude that KG-18 achieved reduced susceptibility to VAN by IS*256*-mediated WalKR overexpression, leading to a markedly thickened cell wall for trapping free VAN molecules with redundant D-Ala-D-Ala targets. In addition, a positively charged surface with lysyl-phosphatidylglycerol and depolarization of wall teichoic acid could contribute to inhibiting cationic daptomycin and VAN antimicrobial activity. Comparative omics approaches in this study strongly suggest that fully complete and annotated genome sequences will be indispensable for characterizing overall VISA phenotype.

## Introduction

*Staphylococcus aureu*s, one of the major nosocomial and community-acquired pathogens, causes a variety of clinical problems, including infections of the skin and soft tissues (Lowy, 1998). Multiple-antibiotic-resistant *S. aureus* continues to be one of the most common pathogens of both hospital-associated and community-associated infections worldwide. Since the 1960s, the prevalence of methicillin-resistant *S. aureus* (MRSA), which has been associated with higher rates of morbidity and mortality than methicillin-susceptible *S. aureus* (MSSA) (Kaye et al., 2004), has increased at a dramatic rate (Maree et al., 2007). Glycopeptides, such as vancomycin (VAN) and teicoplanin (TEIC), are primary and effective antimicrobial drugs for treating MRSA. Currently, the Clinical Laboratory Standards Institute (CLSI) categorizes *S. aureus* as vancomycin susceptible (VSSA) (MIC ≤ 2 μg/mL), vancomycin intermediate resistant (VISA) (4–8 μg/mL), or vancomycin resistant (VRSA) (MIC ≥ 16 μg/mL) (Patel, 2014). The first clinical VISA strain, Mu50 (MIC 8 μg/mL), and the hetero-VISA (hVISA) strain, Mu3 (MIC 2 μg/mL) were isolated in 1996 in Japan (Hiramatsu et al., 1997). However, a retrospective study suggests that reduced susceptibility to vancomycin dates back at least to 1987 in the United States (Jackson and Hicks, 1987).

Vancomycin resistant/hetero-VISA is typically associated with hospitalization, persistent infection, prolonged vancomycin treatment, and/or treatment failure (Casapao et al., 2013). The hVISA phenotype refers to a mixed-cell population in which the majority of cells have little or no resistance to VAN, thus, an hVISA cell population is within the susceptible range when tested with routine methods, but contains a proportion of cells within the VISA range (McGuinness et al., 2017).

A systematic review and meta-analysis of 91 published studies indicated that the prevalence rates of hVISA and VISA were 9.81 and 2.00% in all blood culture samples, respectively (Zhang et al., 2015), and suggested that SCC*mec* II accounted for 48.16 and 37.74% of hVISA and VISA, respectively (Zhang et al., 2015).

Vancomycin intermediate resistant strains exhibit common characteristics including a thickened cell wall (Hanaki et al., 1998; Cui et al., 2003), reduced autolysis, and attenuated virulence (Howden et al., 2011). Several genetic alterations in two-component regulatory systems have been reported to be strongly associated with a VISA phenotype, including mutations in the *vraSR* operon (Mwangi et al., 2007; Cui et al., 2009), *graRS* (Cui et al., 2009), and *walRK* (Howden et al., 2011). A recent review on the molecular characterization of hVISA/VISA summarized a number of variable mutations cataloged in VISA (Hu et al., 2016). These mutations have been identified from various experimental settings under *in vitro* passage, with VAN selection, or comparison of clinical isolates under prolonged VAN (and subsequent other MRSA antimicrobial therapy) treatment.

The mechanism of VAN activity involves binding with the terminal D-alanyl-D-alanine (D-Ala-D-Ala) moieties of murein monomer or peptidoglycan chain, inhibiting transglycosylation and transpeptidation of murein monomers. This VAN binding prevents cross-linking of long peptidoglycan polymers in the bacterial cell wall. This unique VAN inhibitory action implies that the mechanisms underlying the VISA phenotype may be complicated, because a number of identified factors could contribute to the activation of cell wall metabolism through multiple routes, leading to increased cell wall volume, and thus trapping free VAN molecule.

Here, we used multi-omics approaches based on complete genome sequencing, transcriptomics, and proteomics to characterize clinical VISA isolates obtained through prolonged antimicrobial treatment.

## Results

### Clinical Manifestation and Course for VISA Isolates

**FIGURE 1:**
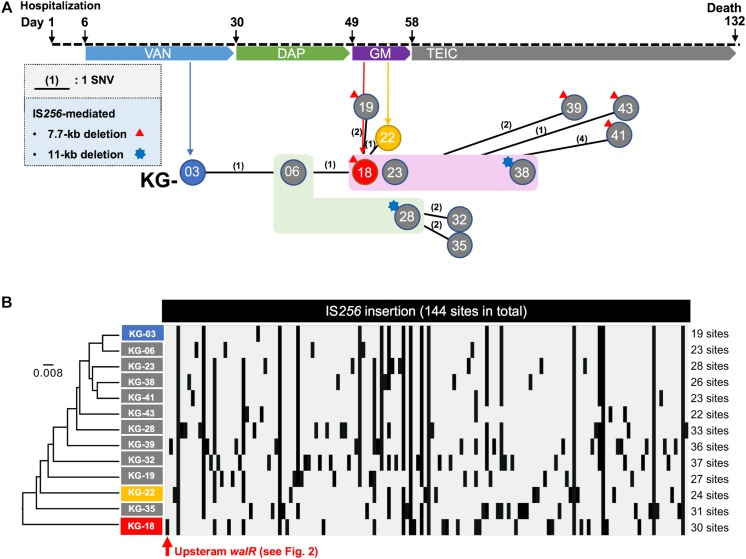
Clinical history of the patient from hospitalization. **(A)** Regimen of antimicrobial therapy, (VAN, DAP, GM, and TEIC). The time points for the thirteen isolates are indicated by circles (KG-xx) below the clinical history. A number in parenthesis indicates core-genome-based SNVs between strains (see all SNVs in Supplementary Table S1). No SNV was found among the strains highlighted in light green (KG-06 and KG-28) or magenta (KG-18, KG-23, and KG-38) backgrounds. In addition to SNVs, strains which showed notable IS*256*-mediated deletion are indicated with a red triangle or blue star at the left shoulder. **(B)** IS*256*-insertion sites in thirteen strains are summarized. The IS*256* insertion profile was analyzed by the UPGMA method.

A patient was hospitalized in 2015 due to reduced platelet count causing systemic bleeding during dialysis treatment for chronic renal failure. At day-4, a phlebitis with fever was observed, and blood cultures revealed MRSA at day-6. VAN treatment was then started. The antimicrobial therapy administered is summarized in Figure 1. There was a pain at the site of the artificial blood vessel, which was considered to be a cause of persistent bacteremia. The patient’s condition included intermittent fever but was stable. However, because the patient refused to have the artificial blood vessel removed, bacteremia persisted, leading to death at day-132.

### Antimicrobial Susceptibility Testing

Antimicrobial susceptibilities of thirteen MRSA isolates were determined (Table 1). Among these, KG-03, isolated during VAN treatment, showed β-lactam resistance but susceptibility to VAN (MIC: 0.5 μg/mL). During the course of VAN treatment for 25 days, moderate fever persisted, and a secondary optimal antibiotic, daptomycin (DAP), was prescribed for the patient. The fever worsened, and, at day-19, gentamicin (GM) was substituted for DAP. During VAN and subsequent DAP treatments, KG-06 showed lower susceptibility to VAN (MIC: 2 μg/mL). Intriguingly, the VISA isolates KG-18 and KG-22 showed markedly reduced susceptibility to glycopeptides (VAN and TEIC) during GM treatment, but were significantly more susceptible to β-lactams (OXA, IPM, MEPM, BIPM, and DRPM). KG-18 and KG-22 isolates exhibited VISA phenotype in addition to reduced susceptibility to DAP (MIC: 2 μg/mL).

**TABLE 1:** Antimicrobial susceptibility of *S. aureus* isolates from a single patient suffering from successive bacteremia during hospitalization.

		**MIC (μg/mL)**																	
**Strain**	**Days after hospitalization**	**VAN**	**TEIC**	**DAP**	**LZD**	**ABK**	**OXA**	**AMP**	**IPM**	**MEPM**	**BIPM**	**DRPM**	**LVFX**	**CPFX**	**MINO**	**EM/CLDM**	**ST**	**QPR/DPR**	**References**
KG-03	24	0.5	1	0.25	2	0.5	>128	16	2	8	8	8	64	64	8	>4/0.5	0.25	1	This study
KG-06	40	2	2	1	2	2	128	16	32	16	32	8	64	64	8	>4/0.5	0.06	1	This study
KG-18	51	**8**	2	**2**	1	4	4	1	<=0.06	0.25	0.25	<=0.06	32	64	8	>4/0.5	0.25	1	This study
KG-19	51	1	1	0.5	1	2	32	16	0.5	2	2	1	16	64	8	>4/0.5	0.25	1	This study
KG-22	54	**4**	4	**2**	1	8	64	8	0.125	2	1	0.5	32	32	4	>4/0.5	0.06	0.5	This study
KG-23	56	1	2	1	1	0.5	64	16	0.125	1	1	0.5	32	64	8	>4/0.5	0.06	1	This study
KG-28	58	1	4	**2**	1	0.5	>128	32	>64	32	64	32	32	32	8	>4/0.5	0.25	1	This study
KG-32	65	2	4	1	2	1	128	8	1	4	4	2	32	64	8	>4/0.5	0.25	1	This study
KG-35	65	**4**	1	1	2	2	64	8	1	2	2	1	32	64	8	>4/0.5	0.06	0.5	This study
KG-38	75	2	2	1	0.5	2	64	8	0.25	1	1	0.5	16	32	4	>4/0.5	0.06	1	This study
KG-39	83	2	2	1	0.5	2	64	8	2	4	8	2	32	32	4	>4/0.5	0.06	0.5	This study
KG-41	99	**4**	4	**2**	1	4	32	4	0.5	2	2	0.5	32	64	8	>4/0.5	0.06	1	This study
KG-43	100	1	4	1	1	0.5	128	8	0.25	0.5	0.5	0.25	32	64	8	>4/0.5	0.25	1	This study
N315	n/a	1	0.5	0.5	2	1	32	16	1	4	4	1	0.5	0.5	0.125	>4/0.5	0.125	1	Kuroda et al., 2001
Mu3	n/a	2	8	1	2	4	>128	16	64	32	64	32	16	32	16	>4/0.5	0.125	0.5	Kuroda et al., 2001
Mu50	n/a	8	8	2	2	4	>128	16	16	8	16	8	32	>16	8	>4/0.5	0.125	0.5	Kuroda et al., 2001
ATCC29213	n/a	1	1	0.5	2	1	<=0.5	0.5	<=0.06	<=0.06	<=0.06	<=0.06	0.5	0.25	0.125	<=4/0.5	0.125	0.5	Type strain

*VAN, vancomycin; TEIC, teicoplanin; DAP, daptomycin; LZD, linezolid; ABK, arbekacin; OXA, oxacillin; AMP, ampicillin; IPM, imipenem; MEPM, meropenem; BIPM, biapenem; DRPM, doripenem; LVFX, levofloxacin; CPFX, ciprofloxacin; MINO, minocycline; EM, erythromycin; CLDM, clindamycin; ST, Sulfamethoxazole-Trimethoprim; QPR/DPR, Quinupristin/Dalfopristin. CLSI breakpoint was adapted.*

### Comparative Genome Analysis of the Sequential Isolates Based on Single Nucleotide Variations and IS256 Insertions

To characterize the genetic features-related to VISA phenotype, draft genome sequences for sequential time-series isolates (KG-03 to -43) were obtained (Table 1), followed by PacBio Sequel SMRT sequencing to determine the complete genome sequences of KG-03, KG-18, and KG-22 (Table 2). All isolates were isogenic, based on multi-locus sequence typing (MLST) ST5, SCC*mec*_type_II (2A), and *spa* type t17639 (repeats: 26-34-34-23-17-12-17-16).

**TABLE 2:** Complete genome sequence information of *S. aureus* strains.

**Strain**	**GC%**	**length (bp)**	**ORFs**	**MLST**	**SCC*mec* type**	***spa* type**	**GenBank ID**
**KG-03**							
chromosome	32.9	2,945,245	2,728	ST 5	type_II (2A)	t17639	AP019542
**KG-18**							
chromosome	33	2,963,149	2,798	ST 5	type_II (2A)	t17639	AP019543
pKG-18	28.6	23,184	24	N/A	N/A	N/A	AP019544
**KG-22**							
chromosome	32.9	2,961,166	2,802	ST 5	type_II (2A)	t17639	AP019545
pKG-22	28.9	21,014	21	N/A	N/A	N/A	AP019546

*N/A, not availabale.*

Core-genome single nucleotide variation (SNV) analysis based on the complete genome sequence of KG-03 as a reference identified 16 SNVs among the thirteen isolates (Figure 1A and Supplementary Table S1). Further strain-specific genetic features were found as two IS*256*-mediated deletions (7.7 kb or 11 kb in Figure 1A, see Supplementary Table S1 for ORFs). One of these deletions, of 7.7 kb (shown as a red triangle in Figure 1A), was observed in KG-18 compared with KG-03. The deleted region included an uncharacterized two-component-system sensor kinase/response regulator, and we therefore speculated that signal transduction may be involved in the VISA phenotype. However, other VAN-susceptible strains (KG-19, -39, -41, and -43) showed an identical deletion, indicating that it might not be associated with VISA phenotype. Notably, mutation of *mprF* (Trp_424_Cys in MprF phosphatidylglycerol lysyltransferase), possibly involving in DAP susceptibility, was observed in KG-22 (DAP MIC: 2 μg/mL) (Table 3), although not in KG-18, which did show reduced DAP susceptibility (MIC: 2 μg/mL).

**TABLE 3:** List of genetic alterations in the genomes of KG-18 and KG-22 compared with KG-03.

**Gene_ID**	**Gene**	**Product**	**KG-03 position**	**KG-03**	**Allele**	**Genetic alteration**
**Common in KG-18 and KG-22**						
KG03_05130	nusG	transcription termination/antitermination protein	576,712	C	T	Pro101Ser
KG03_06380	N/A	ABC transporter permease	714,916	C	T	Pro475Leu
**Specific in KG-18**						
KG03_12270 - KG03_12350	N/A	8 ORFs; threonine aldolase, hypothetical protein, cardiolipin synthase, ABC transporter ATP-binding protein, multidrug ABC transporter permease, two-component sensor histidine kinase, hypothetical protein, thermonuclease	1,334,187-1,341,887	(7,701 bp)	N/A	IS*256*-mediated deletion
**Specific in KG-22**						
KG03_12700	mprF	phosphatidylglycerol lysyltransferase	1,386,141	G	T	Trp424Cys

*N/A, not available. See all comparative genetic alterations including SNVs information in Supplementary Table S1.*

We further investigated the structural variations by IS*256* insertion throughout the genome sequences. Although only two SNVs different between KG-03 and KG-18 were observed, IS*256* insertion profile in KG-18 is clearly distinct from both KG-03 and KG-06 (Figure 1B), suggesting that extensive IS*256* insertion appears to have been generated during DAP treatment.

Our SNV (Figure 1A) and IS*256* insertion (Figure 1B) analysis results did not show consistent molecular phylogeny and evolution. This is because SNVs are generated linearly, with an estimated mean rate of 2.43 × 10^–6^ substitutions per site year^–1^ in *S. aureus* (Duchene et al., 2016), but IS-insertion can be generally induced by SOS-response under stress conditions with antimicrobial selection or environmental insults (Hocquet et al., 2012).

### Transcriptome Analysis for VISA Isolates

#### KG-18-Specific Differential Regulation

To determine the factors involved in VISA phenotype, we focused on three isolates (KG-03, KG-18, and KG-22), that harbored multiple strain-specific chromosomal IS*256* insertions (Figure 1B). Based on genome structure, we speculated that the difference upon IS*256* insertion could contribute to distinct gene expression patterns, leading to VISA phenotype.

**FIGURE 2:**
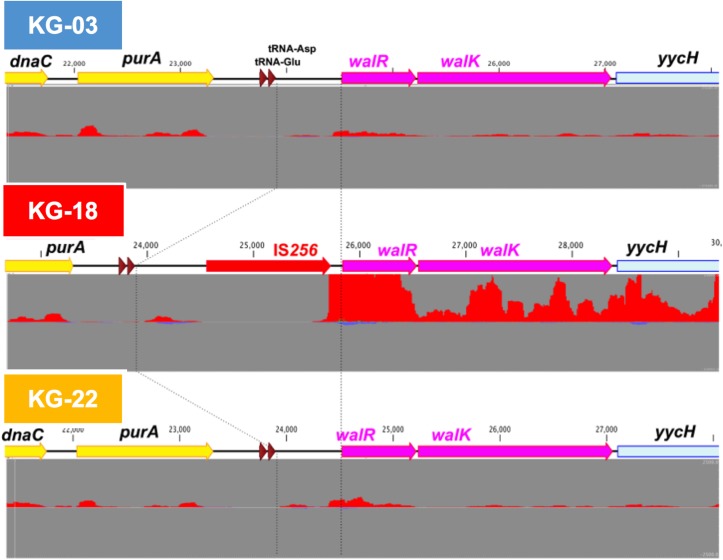
IS*256*-mediated overexpression of the *walKR* operon in KG-18. The IS*256* element was integrated upstream of the *walKR* two-component system in KG-18, but not KG-03 or KG-22. Comparative RNA-Seq revealed markedly increased expression of *walR* (18.6-fold; Table 4). Positive- and negative-strand read mapping is illustrated as red and dark blue, respectively.

Comparative transcriptome analysis revealed that the *walKR* system (*walR, walk, walH, and walI*) in KG-18 showed >10-fold-increased expression compared with VAN-susceptible KG-03 (Table 4). RNA-Seq read mapping showed significant read coverage of the *walKR* system downstream of IS*256* insertion in KG-18 (Figure 2). Such increased expression of the *walKR* system was not observed in KG-22, suggesting that IS*256* insertion could be involved in the overexpression. Both KG-18 and KG-22 showed reduced susceptibility to VAN (8 and 4 μg/mL, respectively), but transcriptome analysis revealed strain-specific gene expression associated with each distinct IS*256* insertion (Figure 1B). The *walKR* system has previously been shown to positively regulate *atlA, lytM, isaA, ssaA*, and *sceD* gene expression (Dubrac et al., 2007). Indeed, this study is also in partial agreement with that previous study, revealing positive regulation of *lytM, isaA*, and *ssaA* homologs, and *sceD* (Table 4). Among those genes, marked overexpression of *lytM* (Glycyl-glycine endopeptidase LytM precursor) and *sceD* (putative transglycosylase SceD precursor) were detected (28.6- and 47.5-fold, respectively). This suggested that these abundant cell-wall-related enzymes could play a role in cell wall structure and integrity pivotal for VAN susceptibility. Other notable upregulated genes were *dltABCD* (D-alanylation of lipoteichoic acid), *mprF* (phosphatidylglycerol lysyltransferase), *ureA/ureB* (urease subunits), and *icaA/icaD* (Poly-beta-1,6-N-acetyl-D-glucosamine synthases), while cell-wall-anchored LPxTG-motif proteins (CWAP) (*spa, coa*, and *clfB*), were downregulated, implying that all notable expression changes are involved in cell wall metabolism or cell surface structure.

**TABLE 4:** Differential gene expression in KG-18 or KG-22 compared with KG-03 by RNA-Seq analysis (≥5-fold difference).

			**Transcripts per million (TPM) value**			**Fold ratio**	
**ORF ID in KG-18**	**Gene**	**Product**	**KG-03**	**KG-18**	**KG-22**	**KG-18/KG-03**	**KG-22/KG-03**
**Increased expression on either KG-18 or KG-22**							
SAKG18_00190	walR^*^	Transcriptional regulatory protein WalR	514.8	9572.5	618.8	18.6	1.2
SAKG18_00200	walK^*^	Sensor protein kinase WalK	149.7	1800.1	106.9	12.0	0.7
SAKG18_00210	walH^*^	WalH protein	189.4	1932.9	130.5	10.2	0.7
SAKG18_00220	*walI*	WalI protein	265.9	2796.6	245.4	10.5	0.9
SAKG18_02750	*lytM*	Glycyl-glycine endopeptidase LytM precursor	152.8	4366.2	859.0	28.6	5.6
SAKG18_03720	–	Peptidase propeptide and YPEB domain protein	120.9	1108.2	165.9	9.2	1.4
SAKG18_05420	*sdrE*	Serine-aspartate repeat-containing protein E precursor	407.9	1086.5	3018.6	2.7	7.4
SAKG18_08380	*dltA*	D-alanine–poly(phosphoribitol) ligase subunit 1	200.7	1398.8	585.9	7.0	2.9
SAKG18_08390	*dltB*	Peptidoglycan O-acetyltransferase	164.7	1203.7	545.9	7.3	3.3
SAKG18_08400	*dltC*	D-alanine–poly(phosphoribitol) ligase subunit 2	733.7	4794.5	2025.2	6.5	2.8
SAKG18_08410	*dltD*	DltD central region	187.4	1331.6	479.2	7.1	2.6
SAKG18_08880	–	65 kDa membrane protein precursor	115.0	1168.9	162.0	10.2	1.4
SAKG18_11080	*pyrC*	Dihydroorotase	97.7	103.4	515.0	1.1	5.3
SAKG18_12240	–	hypothetical protein	4.0	31.6	6.3	7.9	1.6
SAKG18_12650	*mprF*	Phosphatidylglycerol lysyltransferase	80.5	1013.3	253.3	12.6	3.1
SAKG18_14150	–	Phage gp6-like head-tail connector protein	19.9	119.8	21.3	6.0	1.1
SAKG18_21020	*sceD*	putative transglycosylase SceD precursor	210.6	9998.0	1037.8	47.5	4.9
SAKG18_22090	–	65 kDa membrane protein precursor	27.3	171.8	62.3	6.3	2.3
SAKG18_22930	*ureA*	Urease subunit gamma	46.8	456.8	134.9	9.8	2.9
SAKG18_22940	*ureB*	Urease subunit beta	16.0	133.6	44.3	8.4	2.8
SAKG18_23050	–	hypothetical protein	4.2	39.3	9.8	9.4	2.4
SAKG18_23090	*ssaA2*	Staphylococcal secretory antigen ssaA2 precursor	561.0	3716.6	269.5	6.6	0.5
SAKG18_23920	*narT*	putative nitrate transporter NarT	20.7	52.4	136.1	2.5	6.6
SAKG18_23990	*narW*	putative nitrate reductase molybdenum cofactor assembly chaperone	26.5	92.9	134.7	3.5	5.1
SAKG18_24010	*narG*	Respiratory nitrate reductase 1 alpha chain	28.6	52.8	228.8	1.8	8.0
SAKG18_24050	*sirB*	Sirohydrochlorin ferrochelatase	56.4	103.2	503.9	1.8	8.9
SAKG18_25750	*ssaA*	Staphylococcal secretory antigen SsaA precursor	154.0	806.1	330.5	5.2	2.1
SAKG18_26850	–	hypothetical protein	2.4	38.2	15.2	15.7	6.3
SAKG18_27030	–	hypothetical protein	8.0	44.2	17.3	5.5	2.2
SAKG18_27520	*icaA*	Poly-beta-1,6-N-acetyl-D-glucosamine synthase	11.8	67.4	14.1	5.7	1.2
SAKG18_27530	*icaD*	Poly-beta/-1,6-N-acetyl-D-glucosamine synthesis protein	47.9	293.8	132.0	6.1	2.8
**Reduced expression on KG-18 or KG-22**							
SAKG18_01090	*spa*	Immunoglobulin G-binding protein A precursor	7226.3	143.0	797.9	−50.5	−9.1
SAKG18_02290	*coa*	Staphylocoagulase precursor	1204.2	120.7	194.1	−10.0	−6.2
SAKG18_09620	*qoxD*	Quinol oxidase subunit 4	463.8	112.1	91.2	−4.1	−5.1
SAKG18_12120	*hfq*	RNA-binding protein Hfq	38.9	6.8	23.7	−5.7	−1.6
SAKG18_12180	–	hypothetical protein	51.1	8.0	24.0	−6.4	−2.1
SAKG18_12260	–	hypothetical protein	46.4	16.2	9.4	−2.9	−4.9
SAKG18_25270	–	ABC-2 family transporter protein	55.7	18.6	5.6	−3.0	−9.9
SAKG18_27130	*clfB*	Clumping factor B precursor	6970.0	1097.2	4042.4	−6.4	−1.7

**FIGURE 3:**
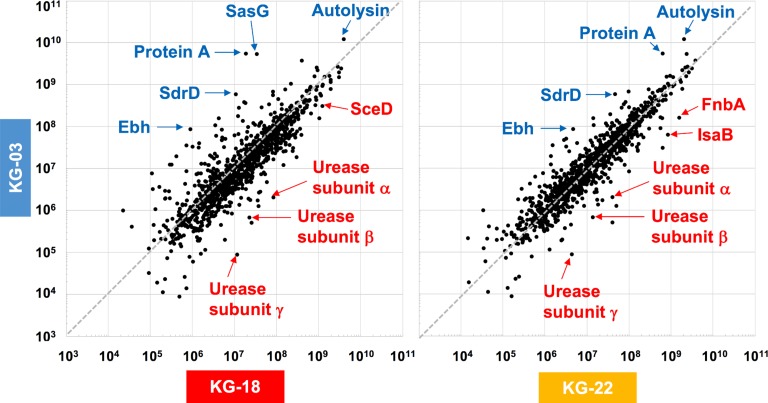
Comparative secretome analysis by data-independent acquisition (DIA) mass spectrometry. Secreted proteins from KG-18 and KG-22 were compared with those of KG-03 as a control strain using nanoLC-MS analysis. The detected MS peaks were analyzed by Scaffold DIA Proteome Software. All detected MS signals are summarized in Supplementary Table S3.

All RNA-Seq data is available in Supplementary Table S2. ^*^See RNA-Seq read-mapping image in Figure 3.

#### KG-22-Specific Differential Regulation

The marked differential expression pattern of KG-22 was partially consistent with that of KG-18, but the extent of this was not apparent (Table 4). Both *lytM* and *sceD* are also significantly upregulated in KG-22 (5.62- and 4.93-fold, respectively). In addition, both *spa* and *coa* expression were downregulated in KG-22 (-9.06- and -6.20-fold, respectively) (Table 4). Notable KG-22-specific upregulation was found in the pyrimidine biosynthesis pathway (*pyrC*), and in the nitrate reduction pathway (*nar* genes), which generates ammonia.

### Secretome Analysis of VISA Isolates

To elucidate the above-mentioned transcriptome analysis further, quantitative proteomics of secreted proteins were conducted using data-independent acquisition (DIA) mass spectrometry (Figure 3). The glycyl-glycine endopeptidase LytM increased by 5.5- and 3.2-fold in KG-18 and KG-22, respectively, compared with KG-03 mass detection, as shown in Supplementary Table S3. Transglycosylase SceD was increased by 3.9- and 0.78-fold in KG-18 and KG-22, respectively, compared with KG-03 mass detection (Supplementary Table S3). Urease subunit proteins were also significantly upregulated in KG-18 and KG-22. Fibronectin-binding protein, FnbA, and immunodominant staphylococcal antigen, IsaB, were increased in KG-22 compared with KG-03 (Figure 3).

In contrast to the LytM and SceD cell-wall-related enzymes described above, bifunctional autolysin (Alt) showed 3.1- and 6.0-fold-reduced expression in KG-18 and KG-22, respectively (Supplementary Table S3). Remarkably, immunoglobulin G-binding Protein A (*spa*) was reduced by 162-fold in KG-18 (Supplementary Table S3), as well as in the transcriptome analysis. Additionally, of note, two CWAPs (SasG and SdrD) and the cell-surface-associated factor Ebh showed reduced expression in KG-18 and KG-22. Taken together, with our transcriptomic observations, DIA mass spectrometry confirmed increased expression of LytM and SceD cell-wall-related enzymes, and reduced expression of autolysin and CWAPs.

### Cell Wall-Related Features (Lytic Enzymes and Wall Susceptibility)

**FIGURE 4:**
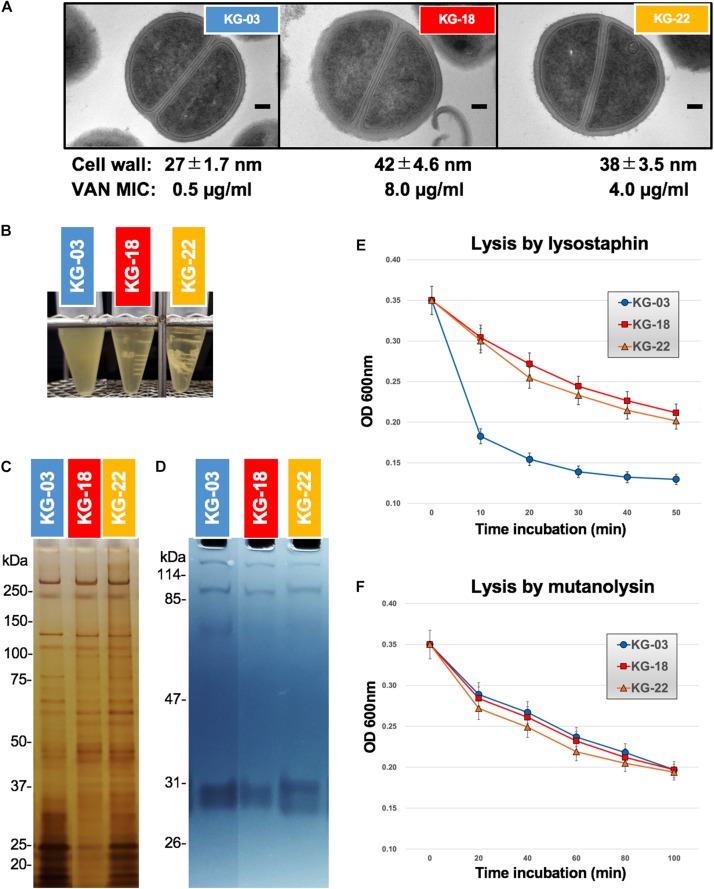
Cell wall-related features. **(A)** Transmission electron microscopy images of representative cells (at least seven cells). Scale bars represent 100 nm. The cell wall thickness, in nanometers (mean ± SD), and VAN MIC are given under each image. **(B)** Slow-growing of KG-18 and biofilm formation of KG-22 in BHI broth under static incubation. **(C)** Silver-staining of cell-surface proteins with SDS-PAGE. **(D)** Auto-lytic enzyme assay by zymography using SDS-PAGE containing heat-killed *S. aureus* NCTC 8325 cells. Lytic resistance for **(E)** lysostaphin or **(F)** mutanolysin was investigated following heat/SDS-inactivation of the tested strains.

Generally, the VISA phenotype has been characterized by increased cell wall volume, observed as a thickened cell wall, leading to increased amounts of VAN-binding target (D-Ala-D-Ala moiety), resulting in reduced susceptibility to VAN (Hanaki et al., 1998). Our transmission electron microscopy (TEM) images suggested that a thickened cell wall was observed in KG-18 and KG-22 (Figure 4A), correlating with VAN MIC, as noted in previous reports. Growth of both KG-18 and KG-22 were significantly slower than KG-03, and KG-22 showed biofilm formation under static incubation (Figure 4B).

To elucidate cell wall lytic activity, we investigated cell wall-associated lytic enzymes using zymography. Although differential expression of surface-associated proteins was observed (silver-staining bands in Figure 4C), cell wall lytic activity around 60-kDa was somewhat reduced in KG-18 and KG-22 (Figure 4D).

To evaluate cell wall integrity, heat/sodium dodecyl sulfate (SDS)-inactivated cells were subjected to commercially available lytic enzymes (lysostaphin and mutanolysin). The results suggested that KG-18 and KG-22 were notably resistant to cell lysis by lysostaphin, which specifically cleaves a staphylococcus-unique pentaglycine chain in the peptidoglycan (Figure 4E). However, there was no apparent difference for mutanolysin, which acts on N-acetylmuramidase cleavage of the β-N-acetylmuramyl-(1→4)-N-acetylglucosamine linkage in peptidoglycan (Figure 4F). This lysostaphin-resistant phenotype suggested that the cell wall of KG-18 and KG-22 appears to be constructed with fewer lysostaphin targets (pentaglycine bridges) than KG-03, resulting in unbridged pentaglycine chains, which could increase the amounts of free D-Ala-D-Ala peptidoglycan ends for trapping VAN.

### DAP Resistance

**FIGURE 5:**
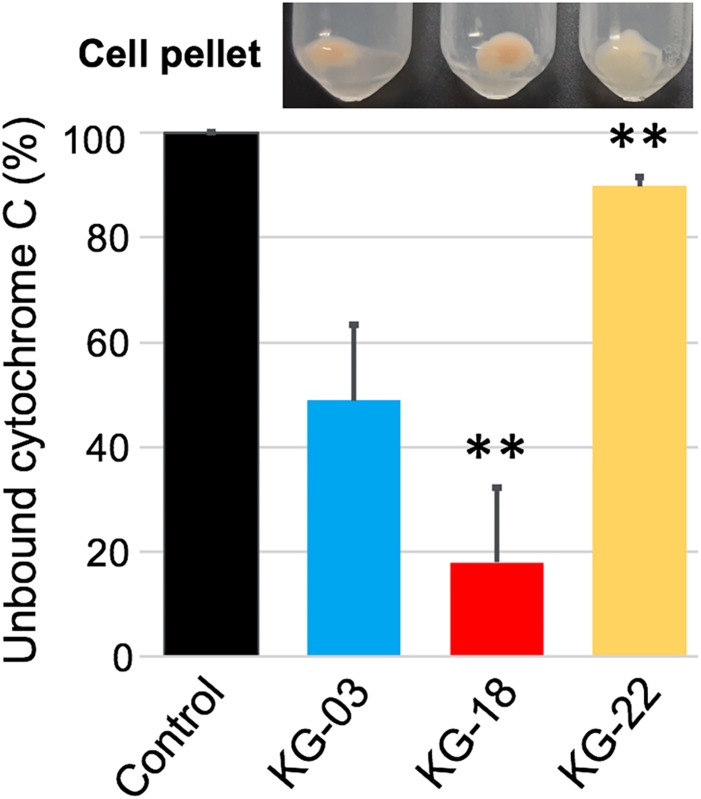
Repulsion assay using a cationic protein, cytochrome *c*, to bacterial cell surface. Control indicates the % value at OD_410__nm_ without bacterial cells. The bar graph shows the % values of unbound cytochrome *c* as supernatant after centrifugation; the residual cell pellet is shown above each bar graph. Values that are significantly different from the values determined for KG-03 are indicated (^∗∗^*P* < 0.01). The assay was performed in triplicate, and error bars show standard deviations.

In addition to reduced susceptibility to VAN, KG-18, and KG-22 showed markedly reduced susceptibility to DAP (Table 1). DAP is a calcium-dependent lipopeptide antibiotic, which has become a standard-of-care agent for treating MRSA infections. The mechanism of DAP resistance has been extensively characterized. Most DAP-resistant isolates possess point mutations in the *mprF* gene, encoding MprF, which synthesizes and translocates positively charged lipid lysyl-phosphatidylglycerol (LysPG) to the outer surface of the cytoplasmic membrane. This leads to a positively charged outer surface, reducing susceptibility to cationic antimicrobial peptides. We found markedly increased MprF expression in KG-18 and KG-22 (12.6- and 3.1-fold, respectively; Table 3). A repulsion assay using a cationic protein, cytochrome *c* (red-colored), showed that KG-18 exhibited rather increased binding of cytochrome *c*, while KG-22 exhibited marked repulsion (Figure 5), suggesting that MprF mutation (Trp_424_Cys, shown in Table 3) could be one of the factors possibly involved in DAP susceptibility.

We also found increased expression of *dltABCD* genes, which is consistent with previous reports revealing that high expression of *mprF* and *dltABCD* could contribute to DAP resistance without *mprF* mutation (Bayer et al., 2016; Ma et al., 2018; Sabat et al., 2018).

Regarding DAP resistance-mediated re-sensitization to β-lactams (a seesaw effect) (Renzoni et al., 2017), KG-18 and KG-22 showed remarkably increased susceptibility to all tested β-lactams (OXA, AMP, IPM, MEPM, BIPM, and DRPM). The transcription level of *mecA* was reduced by 1.87- and 2.26-fold in KG-18 and KG-22, respectively (Supplementary Table S2); thus, the reduced expression of fundamental β-lactam resistance factors might be associated with the seesaw effect.

## Discussion

In this study, we obtained VISA isolates through sequential antimicrobial therapy in a single patient. Comparative complete genome sequences and transcriptomic and proteomic analyses demonstrated that IS*256* insertion is involved in overexpression of the WalKR two-component regulatory system, leading to differential gene expression related to cell wall integrity (*lytM* and *sceD*), surface charge (*mprF* and *dltABCD*), poly-glucosamine biofilm formation (*icaAD*), and downregulation of CWAPs (*spa, coa*, and *clfB*). In general, whole genome sequencing can identify notable genetic features associated with phenotypes of interest. However, in this study, analysis of genome-wide SNVs using only draft genome sequence was not able to unveil VISA phenotype-related genetic alterations due to extensive IS*256*-mediated structural variations (Figure 1B).

The contribution of the WalKR system has been well evaluated previously (Dubrac et al., 2007). It is known to play an essential role in glycopeptide susceptibility, and various genetic alterations have been identified including mutations or differential expression of the system. A number of previous *in vitro* studies (Shoji et al., 2011; Hu et al., 2015; Wang et al., 2016; Peng et al., 2017) and clinically relevant isolates (Howden et al., 2011; Hafer et al., 2012) have demonstrated that non-synonymous mutations of either *walk* or *walR* could contribute to the VISA phenotype. In addition, as well as KG-18, IS*256*-mediated *walKR* overexpression associated with a potential hybrid promoter has been reported in a clinical VISA isolate (SA137/93A; VAN Etest: 8 μg/mL) (Jansen et al., 2007). Indeed, both clinical isolates [KG-18 and SA137/93A (Jansen et al., 2007)] showed an identical IS*256* insertion site upstream of *walKR* 5′ UTR at nucleotide position -59 from the initiation codon (ATG).

In contrast, McEvoy et al. (2013) reported the opposite finding in relation to *walKR* expression. Based on qRT-PCR, their *in vitro* VAN-selected VISA isolates showed 50% reduced *walKR* expression, following IS*256* insertion at the -38 or -50 nucleotide positions. However, the magnitude of the difference they showed was much lower than we found in this study (>10-fold increase, based on RNA-seq analysis). Interestingly, our results were consistent with those of McEvoy et al. in relation to increased expression of *ssaA and sceD* (Table 4), indicating that similar expression of genes involved in cell-wall metabolism could play a key role in the VISA phenotype.

WalKR transcriptional regulation and its possible regulons have been described (Dubrac et al., 2007; Howden et al., 2011) and reviewed (Hu et al., 2016), but are not fully understood, because the actual stimulus for the WalK two-component sensor kinase has not been identified. More specifically, the mechanism by which the wild or mutated WalKR system is activated through phosphorylation, mediating its signal transduction, is insufficiently characterized. Likewise, the effects of WalKR overexpression, in *in vitro* experiments or clinical isolates *in vivo*, might depend on the strain-specific genetic background, because overexpression might cause either significantly high signal transduction or a dominant-negative effect leading to shutting down of correct WalKR signal transduction. Thus far, the actual stimuli or factors for WalKR have not been identified; indeed, this study suggested that WalKR overexpression appears to positively regulate at least two genes related to cell wall transglycosylase activity (*lytM* and *sceD*), while negatively regulating immunoglobulin G-binding Protein A (*spa*) and coagulase (*coa*) (Table 3).

In common with our study, another previous proteomics analysis showed a high level of SceD (corresponding to SAV2095 in Mu50) in a VISA Mu50 strain (Drummelsmith et al., 2007). The same study used real-time RT-PCR to further reveal that *sceD* mRNA level was significantly induced in all VISA isolates, consistent with our observation of increased levels of *sceD* transcript (Table 3) and SceD protein (Figure 4 and Supplementary Table S3).

Our proteomic MS analysis demonstrated expression of secreted cell-surface proteins consistent with our transcriptomic analysis. Several CWAPs were markedly reduced, correlating inversely with VAN MIC (Figure 4). Intriguingly, a previous VISA population genomics study characterizing a heterogeneous population of VISA-related mutations with deep sequencing under antimicrobial therapy in single patient, reported that 7 out of 20 genes encoding CWAPs showed statistically significant mutation, which was far more than expected by chance (Rishishwar et al., 2016). Along with our study, these observations suggested that mutation and reduction of CWAP could preserve redundant cross-linking ends of the peptidoglycan cell wall layer, exposing more D-Ala-D-Ala binding sites for trapping free-VAN molecules.

**FIGURE 6:**
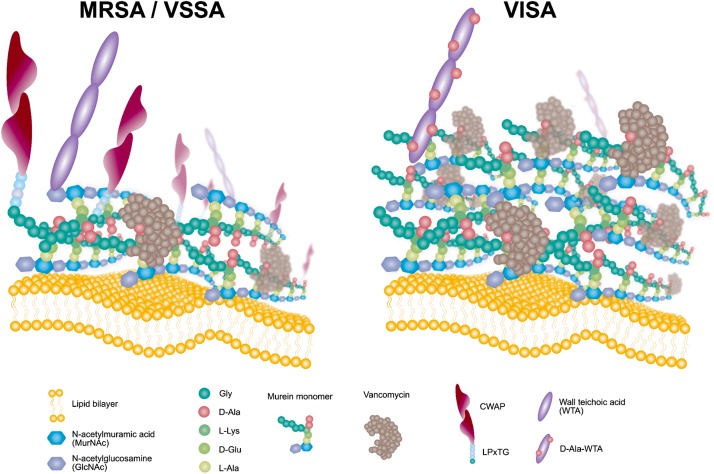
Schematic model of the VISA phenotype displaying redundant D-Ala-D-Ala targets for VAN trapping.

The *narT*, *narW*, and *narG* genes were upregulated in KG-22 compared with KG-18 (Table 4). These genes encode one of the subunits for nitrate reductase that produces nitrite from nitrate, leading to ammonia. Such overexpression of nitrate reductase could provide sufficient amounts of ammonia for *de novo* synthesis of amino acids, in particular L-glutamine. L-glutamine is one of the essential resources for N-acetylglucosamine (GlcNAc), and subsequent *de novo* synthesis of N-acetylmuramic acid (MurNAc). Murein monomer is synthesized by MurABCDEFYG enzymes under sufficient supply, leading to a thickened cell wall (Figures 4A, 6). Increased expression of urease (Table 4 and Figure 3) could also support the supply of ammonia.

We expected that a genome comparison between KG-03 and KG-22 should highlight some factors involved in the reduced susceptibility to VAN, but we could not be confident in addressing pivotal genetic alterations even by performing analyses of genome-wide SNVs and IS*256*-related structural variations. In contrast, secretome analyses suggested KG-22-specific features, and specifically that FnbA and IsaB were increased in KG-22 compared to those in KG-03 (Figure 3). In addition, we noticed unique biofilm features of KG-22 (Figure 4B), such as increased cell-wall related proteins, which might contribute to biofilm formation (Mishra and Horswill, 2017), possibly leading to reduced susceptibility to VAN, at least in part.

In addition to the VISA phenotype, KG-18 and KG-22 showed reduced susceptibility to DAP, and our results suggested that the dose-dependent upregulation of *mprF* transcription (Table 3) could contribute to the increased MIC levels (Table 1). Unlike previous studies reporting that non-synonymous mutations of hot spots in the bifunctional domain of *mprF* (frequently reported in Thr_345_Ala) (Bayer et al., 2015), we did not identify any nucleotide mutations in KG-18, but we did identify a potential non-synonymous mutation (Trp_424_Cys) in KG-22, and a subsequent repulsion assay suggested that KG-22 exhibited significant repulsion to cationic cytochrome *c* (Figure 5). We speculated that the total effects of genetic alterations and the extent of *dltABCD* and *mprF* expression should lead to DAP susceptibility in the end. KG-22 exhibited moderately increased *dltABCD* and *mprF* expression (3-fold and 3.1-fold, respectively, in Table 4) compared to the increase in KG-18 (7-fold and 12.6-fold, respectively, in Table 4). Thus, the non-synonymous mutation (Trp_424_Cys) in MprF of KG-22 might contribute to the marked repulsion of cationic DAP (Figure 5) to explain the remaining extent of reduced susceptibility to DAP.

A recent study showed that the non-synonymous mutation Thr_345_Ala of *mprF* was associated with reduced susceptibility to DAP, whereas the recombinant mutant harboring Thr_345_Ala-type MprF does not alter cellular LysPG or cell-surface charge (Ernst et al., 2018). Thus, the *mprF* mutations may have other effects relating to DAP susceptibility. Besides *mprF* mutations, a number of other reports found marked induction of *mprF* and *dltABCD* transcription in strains with reduced DAP susceptibility (Yang et al., 2009; Kang et al., 2017; Ma et al., 2018), consistent with our observations (Table 3). Significant *dltA* induction generates more D-alanylation to wall teichoic acid (D-ala-WTA), leading to reduced DAP susceptibility in an *S. aureus* clinical isolate (Bertsche et al., 2011). It appears that such *mprF*- and *dlt-*mediated dual enhancements of positive surface charge *via* LysPG and D-ala-WTA, respectively, may be the fundamental mechanism to confer reduced susceptibility to DAP in *S. aureus.*

Fatty acid composition of *S. aureus* cells affects membrane fluidity and negative net surface charge (Goncalves and de Carvalho, 2016) that could contribute to a repulsion mechanism toward the negatively charged (at neutral pH) teicoplanin molecule, but should have the opposite effect toward cationic (at neutral pH) vancomycin, which could be attracted to a more negative membrane. In this study, KG-22 exhibited significant repulsion to cationic molecule (Figure 5), indeed, hVISA/VISA strains have been found to present in common an increased positive cell wall charge responsible for the repulsion of vancomycin (Cafiso et al., 2012).

A previous case report in Japan identified VISA isolates with DAP susceptibility reduced from 0.125 μg/mL (TD1 strain) to 1.0 μg/mL (TD3 strain) without any mutations of *mprF* (Yamaguchi et al., 2015). The antimicrobial regimen used in that study (VAN for 12 days, and subsequently DAP for 30 days) was similar to that given to the patient in this study (VAN for 24 days, and subsequently DAP for 19 days; Figure 1). Both VAN and DAP are categorized as positively charged polypeptide antibiotics that work mainly around the cytoplasmic outer surface, suggesting that the genetic alterations upon possible cross-resistant mechanisms might be generated by similar molecular properties.

## Conclusion

Comparative omics approaches demonstrated that the VISA clinical isolate KG-18 achieved reduced susceptibility to VAN by IS*256*-mediated WalKR overexpression. This mediated the induction of cell-wall-related enzymes (SceD and LytM) and reduced CWAPs (Protein A), leading to a markedly thickened cell wall and redundant free D-Ala-D-Ala targets for VAN binding (Figure 6). In addition, positively charged membrane linked to dysregulation of LysPG by MprF and surface depolarization of WTA by DltABCD could contribute to inhibition of the cationic antimicrobial killing action of DAP, as well as that of the cationic glycopeptide VAN.

## Materials and Methods

### Ethical Approval and Consent to Participate

The study protocol was approved by the National Institute of Infectious Diseases in Japan (Approval No. 642). It was conducted according to the principles of the Declaration of Helsinki, in compliance with the Law Concerning the Prevention of Infections and Medical Care for Patients of Infections of Japan. The ethical committee waived the need for written consent regarding the research into bacterial isolates. The personal data related to the clinical information were anonymized, and our procedure is not to request written consent for all patients suffering from bacterial infections.

### Bacterial Strains

*S. aureus* strains KG-xx were sequentially isolated from human blood specimens during the patient’s persistent bacteremia (Table 1).

### Antimicrobial Susceptibility Testing

The MIC for each antimicrobial used was determined by the broth-dilution method using the CLSI criteria (Clinical and Laboratory Standards Institute [CLSI], 2018). Abbreviations for antimicrobial agents are defined in Table 1.

### Whole-Genome Sequence Analysis

Whole-genome sequencing was carried out as described previously (Sekizuka et al., 2018). Briefly, bacterial cell suspension was inactivated with phenol/chloroform, followed by bead-beating for 10 min by vortexing in ZR BashingBead lysis tubes (Zymoresearch, Irvine, CA, United States). The cell lysate was then purified using a Qiagen DNA purification kit (Qiagen Carlsbad, CA, United States). A DNA-seq library was constructed using a QIAseq FX DNA Library Kit (Qiagen). Whole-genome sequencing was performed using the Illumina NextSeq 500 platform (Illumina, San Diego, CA) with the 300-cycle NextSeq 500 Reagent Kit v2 with paired-end read sequencing (2 × 150-mer; median coverage: >50×).

The complete genome sequences (KG-03, KG-18, and KG-22 strains) were determined by long-read sequencing using a PacBio Sequel sequencer (Sequel SMRT Cell 1M v2 [4/tray]; Sequel Sequencing Kit v2.1; insert size approximately 10 kb). Purified genomic DNA (∼200 ng) was used to prepare a SMRTbell library using a SMRTbell Template Prep Kit 1.0 (PacBio, Menlo Park, CA, United States) with barcoded adaptors according to the manufacturer’s instructions.

Sequencing data were produced with more than 100-fold coverage and assembled using the following programs: Canu version 1.4 (Koren et al., 2017), Minimap version 0.2-r124 (Li, 2016), Racon version 1.1.0 (Vaser et al., 2017), and Circlator version 1.5.3 (Hunt et al., 2015). Error correction of tentative complete circular sequences was performed using Pilon version 1.18 with Illumina short reads (Walker et al., 2014).

Annotation was performed using the DDBJ Fast Annotation and Submission Tool (DFAST) (Tanizawa et al., 2018), and NCBI-BLASTP/BLASTX. Antimicrobial resistance genes were identified by homology searching against the ResFinder database (Zankari et al., 2012). MLST was performed using SRST2 (Inouye et al., 2014). SCC*mec* typing was performed using SCCmecFinder 1.2 (Kaya et al., 2018). Typing of *spa* was performed using spa Typer 1.0 (Bartels et al., 2014). Virulence factors for *S. aureus* were predicted using VirulenceFinder analysis (Kleinheinz et al., 2014).

### Comparative Genome Sequence Analysis

All draft and complete genome sequences of *S. aureus* strains isolated from the patient were compared using bwaMEM to map reads to the *S. aureus* KG-03 complete genome sequence (GenBank ID: AP019542) as a reference. Repeat regions were identified and excluded from further core-genome phylogenetic analysis using NUCmer (Kurtz et al., 2004), as these SNVs are considered unreliable. No recombination was identified by Gubbins prediction software (Croucher et al., 2015). The core genome SNV analysis was performed using the maximum likelihood phylogenetic method with FastTree v2.1.10. All comparative genetic alterations including SNV information are available in Supplementary Table S1. IS*256*-insertion sites were identified using a previously described procedure (Sekizuka et al., 2015). The IS*256* insertion profile was analyzed using the unweighted pair group method with the arithmetic mean (UPGMA) method.

### RNA-Seq Transcriptome Analysis

Bacterial cells grown to logarithmic stage (OD_600__nm_ = 0.4) were harvested by brief centrifugation. The cell pellet was suspended with TE and phenol/chloroform, followed by bead-beating for 10 min by vortexing in ZR BashingBead lysis tubes (Zymoresearch, Irvine, CA, United States). Total RNA was purified from the cell lysate using a miRNeasy Mini Kit (Qiagen) according to the manufacturer’s instructions. RNA-Seq libraries were prepared using the ScriptSeq v2 RNA-Seq Library Preparation Kit (Illumina) according to the manufacturer’s instructions. The RNA-seq libraries were sequenced as a single-end 151-mer on a NextSeq 500 sequencer using the NextSeq 500/550 Kit v2 (Illumina). The transcriptome analysis was performed using CLC Genomics Workbench 10.1 software (Qiagen K.K.). ORFs with transcripts per million (TPM) and false discovery rate (FDR)-normalized *p*-values below 0.05 were considered significant. All RNA-Seq original data are available in Supplementary Table S2.

### Data-Independent Acquisition Mass Spectrometry

For comparative secretome analysis, 20 mL of culture supernatant was grown in brain heart infusion (BHI) broth to logarithmic stage (OD_600__nm_ = 0.6), and concentrated to a 500 μL vol. (40-fold concentration) using AmiconUltra-4 mL (3 kDa m.w. cutoff) (Millipore, MA, United States), the tested loading samples were ready to be analyzed for DIA mass spectrometry by the Kazusa DNA Research Institute (Kisarazu, Japan). Briefly, 20 μg of secreted total protein was reduced by dithiothreitol, followed by iodoacetamide alkylation at cysteine residues, and digested by Lys-C protease and trypsin. The digested peptides were purified, followed by nanoLC-MS analysis with a nanoLC: UltiMate 3000 RSLCnano LC System (Thermo Fisher Scientific, MA United States) and MS: Q Exactive HF -X (Thermo Fisher Scientific). The detected MS peaks were analyzed by Scaffold DIA Proteome Software using coding sequences in KG-18 as references.

### Zymogram Assay for the Detection of Peptidoglycan Hydrolases

The bacterial cells were grown in 20 mL BHI broth to logarithmic stage (OD_600__nm_ = 0.6), and harvested by brief centrifugation. The cell pellet was suspended with 1 mL of 4% SDS, and rotated at 200 rpm for 1 h at 4°C. The cell suspension was centrifuged, and the supernatant containing peptidoglycan hydrolases was filtered through 0.45 μm pore-size membrane. Approximately 1 mL of filtered supernatant was concentrated to 50 μL vol. (20-fold concentration) using AmiconUltra-4mL (3 kDa m.w. cutoff) (Millipore). The tested loading samples were then ready to be analyzed.

Zymography substrate (inactivated whole cells) was prepared from *S. aureus* NCTC 8325 strain in 200 mL BHI broth cultivation. The cell pellet was suspended with 4% SDS and inactivated at 65°C for 2 h, followed by a 4% SDS wash to prepare white color cell suspension. The cell suspension was then added to final 12.5% SDS-PAGE solution (40 mL in total), and polymerized in a gel cassette (8 × 8 cm square). The prepared zymography gel was incubated at 65°C for 2 h to inactivate ammonium persulfate to avoid inactivating loading proteins (peptidoglycan hydrolases). Five microliters of each tested loading sample was loaded into the zymography gel, and run at a constant 20 V at 4°C for >8 h. The gel was washed with 100 mL × 5 of deionized water, followed by immersion with phosphate buffered saline (PBS) and incubation at 37°C until observation of lytic enzyme activity.

### Lytic Enzyme Susceptibility Test

The bacterial cells were grown in 20 mL BHI broth to logarithmic stage (OD_600__nm_ = 0.6), and harvested by centrifugation. The cell pellet was suspended in 20 mL of 4% SDS, and inactivated at 65°C for 2 h. The inactivated cell suspension was centrifuged, and the cells were washed with deionized water three times. The washed cell pellet was suspended with PBS to reach 0.4 OD_600__nm_, and then tested for susceptibility to commercially available lysostaphin (*E. coli* recombinant, Prospec, Ness-Ziona, Israel) or mutanolysin (recombinant, A&A Biotechnology, Gdynia, Poland) lytic enzyme. Lysostaphin and mutanolysin were added to 1 mL of the tested cell suspension at a final concentration of 8 μg/mL and 0.2 U/mL, respectively, followed by incubation in a plastic cuvette at 30°C at 120 rpm rotation. The reduction in OD by cell lysis was measured using a DU730 spectrophotometer (Beckman Coulter, CA, United States).

### Transmission Electron Microscopy

Transmission electron microscopy images were obtained by HANAICHI UltraStructure Research Institute (Kyoto, Japan). Cell wall thickness in nanometers (mean ± SD) was measured from representative cell images (at least seven cells).

### Repulsion Assay of Cationic Cytochrome c

Differences in the bacterial capacity to repulse cationic protein was determined by comparing the levels of binding of the red-colored cationic protein cytochrome *c* from bovine heart (SigmaAldrich, St. Louis, Missouri, United States) as described previously (Ernst et al., 2018) with some modifications. Cytochrome *c* solution was diluted from 0.25 mg/ml (Ernst et al., 2018) to 0.05 mg/ml in this study to bind a half of cytochrome *c* to parental strain KG-03 (Figure 5), because the original concentration of cytochrome *c* was overloaded to the tested bacterial cell density to determine the repulsion potential.

## Data Availability

The complete genomic sequences and annotations of *S. aureus* strains KG-03, KG-18, and KG-22 were deposited in the public database DDBJ (GenBank ID: AP019542, AP019543, and AP019545, respectively). The short- and long-read DNA sequences have been deposited in the DDBJ Sequence Read Archive under the accession number DRA008118 (BioProject: PRJDB8056; BioSample: SAMD00164374–SAMD00164386, and Experiment: DRX161303–DRX161321).

## Author Contributions

JO and TO collected the clinical specimens and isolated the strain from the patient. MK and TS performed the genome sequencing and the comparative genome analysis of *S. aureus* strains. HM and HH contributed to the characterization of the clinical isolates. MK wrote the manuscript.

## Conflict of Interest Statement

The authors declare that the research was conducted in the absence of any commercial or financial relationships that could be construed as a potential conflict of interest.
